# Hepatic inflammatory pseudotumor-like follicular dendritic cell tumor with hepatic lymphoma history: A case report and literature review

**DOI:** 10.1097/MD.0000000000027392

**Published:** 2021-10-01

**Authors:** Jiang Li, Hai-su Tao, Dong Chen, Zhi-yong Huang, Er-lei Zhang

**Affiliations:** a Hepatic Surgery Center, Tongji Hospital, Tongji Medical College, Huazhong University of Science and Technology, Wuhan, PR China; b Department of Hepatobiliary Surgery, The First Affiliated Hospital, College of Medicine, Shihezi University, Shihezi, Xinjiang, PR China; c Department of Pathology, Tongji Hospital, Tongji Medical College, Huazhong University of Science and Technology, Wuhan, PR China.

**Keywords:** case report, follicular dendritic cell sarcoma, hepatic non-Hodgkin lymphoma, inflammatory pseudo tumor-like, liver resection

## Abstract

**Rationale:**

Hepatic inflammatory pseudotumor (IPT)-like follicular dendritic cell (FDC) sarcoma is a very rare disease. Till now, only 19 cases were reported in the English literature. However, the coexistence of IPT-like sarcoma and non-Hodgkin lymphoma (NHL) in the same patient has never been reported. In this report, we presented a case of hepatic IPT-like FDC with hepatic NHL history of which both were successfully resected.

**Patient concerns:**

We reported a case of a 47-year-old male patient who presented with right upper abdominal discomfort. Nineteen years ago, he underwent liver resection of segment VII for hepatic NHL (B-cell lymphoma). He had a history of chronic hepatitis B virus infection. Serum alpha fetoprotein level was normal. However, imaging studies revealed a well-circumscribed, solid mass in the right hepatic lobe, he came back to the clinic because he was worried about a recurrence of the B-cell lymphoma.

**Diagnoses:**

Based on the patient's past medical history and magnetic resonance imaging results, and he was diagnosed as hepatocellular carcinoma or hepatic NHL preoperatively.

**Interventions:**

Right hemi-hepatectomy was performed on this patient.

**Outcomes:**

Histological report showed features of a mixture of chronic inflammatory cells and variable amounts of spindle cells. Also, immuno-histo-chemical studies demonstrated that all the tumor cells showed strong nuclear in situ labeling for EBV-encoded small RNAs and strongly positive stainings with CD21 and CD35. The patient tolerated the surgery well, recovered smoothly and he was discharged on postoperative day 7 (day 7). The patient is still disease free after a follow-up of over 50 months.

**Conclusions:**

To our knowledge, this is the first report demonstrating hepatic IPT-like FDC sarcoma in a patient with primary hepatic NHL history. In regards to treatment, complete surgical resection should be performed and would acquire excellent long-term outcomes.

## 1 Introduction

Follicular dendritic cell (FDC) sarcoma is a very rare disease with more than half of the cases occurring in lymph nodes. Extra-nodal follicular dendritic cell tumors mainly arise from intra-abdominal organs such as liver and spleen, which display an aggressive clinical course.^[1]^ Histologically, there are 2 types of FDC tumor: conventional and inflammatory pseudo-tumor (IPT)-like variant.^[2]^ IPT-like FDC sarcoma contains a few spindle cells with prominent lymphocytes and plasma cell infiltration with some neoplastic cells resembling Hodgkin cells.^[3]^ Hepatic IPT-like FDC sarcoma has been rarely reported in recent years, even though it has been receiving increased attention since its first description in 1996 by Selves et al.^[4]^ Clinico-pathological characteristics of hepatic IPT-like FDC sarcoma have not been fully understood.^[1,5]^ So far, <20 cases of hepatic IPT-like FDC sarcomas have been published in the literature.^[3,4,6–17]^ Due to these morphologic features, IPT-like FDC tumors are commonly misdiagnosed as inflammatory lesions and occur almost exclusively in the liver or spleen with a slight female predominance. Recent studies indicated that these tumors had a risk of recurrence and metastases.^[1,5]^ The origin of tumor cells, causes of the disease, and the ambiguity of diagnosis are still unknown. Hepatic IPT-like FDC sarcomas, which is different from other extra-hepatic FDC tumors, have always been shown to be associated with the Epstein-Barr virus (EBV) infection. EBV infection is considered as one of the most important etiologies of this tumor. Almost 100% of the IPT-like FDC tumors have always been proven to be associated with the EBV infection.^[18]^ Although hepatic IPT-like FDC sarcoma is extremely uncommon, most published cases showed typical histological characteristics, consistent with EBV infection and had a good prognosis after surgical resection of the tumor.^[1,19]^

The distinction of IPT-like FDC sarcoma from other tumors is very challenging and such tumors are commonly misdiagnosed as inflammatory lesions. The regression of the tumor size, either spontaneously or after treatment with anti-inflammatory agents may increase the likelihood diagnosis of IPTs.^[20,21]^ The tumor had no enhancement in all 3 phases after the injection of contrast materials in contrast-enhanced ultrasonography.^[22]^ Central septations, calcification, necrosis, or hemorrhage may be present.^[13]^ Some atypical hepatic IPT-like FDC sarcomas may present with arterial enhancement and are hard to differentiate from hepatocellular carcinomas (HCCs). The definite diagnosis of hepatic IPT-like FDC sarcoma should rely on histopathology. The tumor is a mixture of lymphocytes, storiform, and fascicular arrangement of spindle cells which are positive for at least one of the markers for FDCs including CD21, CD23, CD35, Fascin, Clusterin, CXCL13, and epidermal growth factor receptor.^[23]^

Primary malignant lymphoma of the liver is also a rare disease. In addition, the occurrence of hepatic IPT-like FDC sarcoma and non-Hodgkin lymphoma (NHL) in the same patient has never been reported. In this report, we presented a case of hepatic IPT-like FDC sarcoma with hepatic NHL history, both of them received liver resection.

## 2 Case report

**Figure 1:**
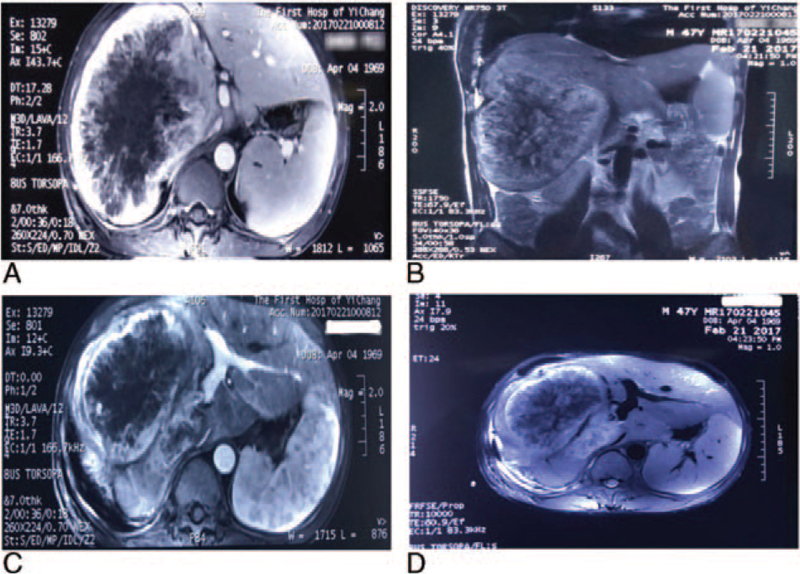
A–B: MRI of the up-abdomen showed a 19 × 15 × 13 cm lesion occupied most of the right liver. No evidence of other abdominal lesions. A MRI image in the axial plane (A, C–D) and in the coronal plane (B). MRI = magnetic resonance imaging.

We evaluated a 47-year-old man with right upper quadrant abdominal pain and no other associated symptoms. He had a history of Hepatitis B virus (HBV) infection and no Hepatitis C infection. Abdominal magnetic resonance imaging (MRI) scan from the First Hospital of Yi Chang revealed a 19 × 15 × 13 cm tumor in the right lobe of the liver. The right portal and hepatic veins were not visualized, inferior vena cava (IVC) was compressed (Fig. 1A–D). In 1999, this patient underwent hepatic segmentectomy in our hospital due to NHL (B-cell lymphoma) of the liver. He had no fever, anemia, weight loss, or constitutional symptoms. Abdominal ultrasound disclosed a 17.5 × 14.2 × 13.6 cm mass in the right lobe of the liver (not available). He had a history of HBV infection, however, serum alpha fetoprotein, carcinoembryonic antigen, and carbohydrate antigen (CA) 199 were all in the normal range and other laboratory tests were unremarkable. HCC or hepatic sarcoma was suspected according to the above findings. The indocyanine green retention rate at 15 minutes was 4.2%. The patient agreed to perform right hepatectomy. The abdomen was carefully inspected during operation and no other lesions were noted within the mesentery, small bowel, or spleen. Frozen sections of suspicious lymph nodes were tested intraoperatively and were negative for metastases. The right lobe of the liver was notably atrophied, likely secondary to tumor thrombosis of the right portal vein, which was showed on MRI imaging. The patient tolerated the surgery well, recovered smoothly, and he was discharged on postoperative day 7.

Surgical specimen was collected for histological examination, processed with standard paraffin technique, and stained with routine hematoxylin-eosin (HE) staining procedures. Immuno-histo-chemical (IHC) and in situ hybridization analyses were performed on a 4-μm thick section. The patient did not receive any adjuvant therapy, and was still alive without tumor recurrence for >50 months after liver resection.

## 3 Histological findings

**Figure 2:**
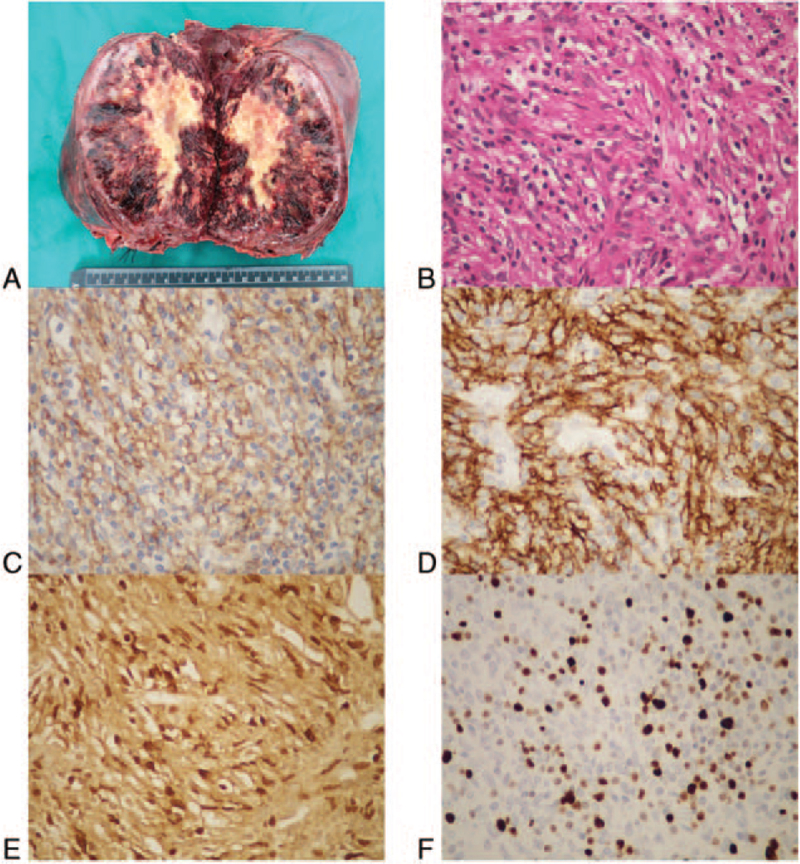
Macroscopic and microscopic findings of the excised tumor. A: The tumor measured 20 × 18 × 15 cm and was solid. B: Microscopically, the hepatic tumor comprised spindle-like cells forming a vaguely storiform pattern and diffuse sheets with the infiltration of small lymphocytes and plasma cells (HE). C–D: Immunohistochemical staining for CD21 and CD23, a marker for follicular dendritic cells, showed that spindle-like cells were positive for CD21 (C) and CD35 (D). E: Positive stains of EBV-encoded nuclear RNAs on the most of spindle cells by in situ hybridization. F: One fourth of the tumor cells were positive for antigen KI67. (Original magnification ×400).

The excised mass measured 20 × 18 × 15 cm, which was well delimited from the surrounding liver parenchyma. The surgical margins were free of tumor, with areas of necrosis and hemorrhage in the center of the tumor (Fig. 2A). Microscopically, the hepatic tumor comprised of spindle-like cells forming a vaguely storiform pattern with blunt cellular border and eosinophilic cytoplasm (Fig. 2B, hematoxylin-eosin stain, ×200) and diffuse sheets with the infiltration of small lymphocytes and plasma. The nuclei were oval to elongated with small or inconspicuous nucleoli. The cell boundary was indistinct and mitoses of the tumor cells were negligible.

## 4 IHC studies

The resected tissue was fixed in formalin, embedded in paraffin, and was cut into 4 μm slice for further analysis. For IHC studies, antigen retrieval by microwaving in sodium citrate buffer was performed. As a result, spindle-like cells were found to express CD21 (Fig. 2C) and CD35 (Fig. 2D) but not CD23, SMA, Desmin, ALK1, and S-100. Also, no chromosomal abnormality was observed. EBV-encoded nuclear RNA in situ hybridization was performed, showing positive signals on the spindle cells (Fig. 2E) but not on the surrounding lymphocytes. The positive stain rate of antigen KI67 in spindle cells was about 25% (Fig. 2F). Based on these findings, a hepatic FDC tumor was diagnosed. The postoperative period was unremarkable and the patient received regular follow-up after discharge. Written informed consent was obtained from the patient for publication of this case report and any accompanying images. This study was conducted in accordance with the Helsinki Declaration and approved by the Medical Ethics Committee of Tongji Hospital Affiliated to Huazhong University of Science and Technology.

## 5 Discussion

FDC tumors are very rare neoplasms that mainly arise in lymph nodes and about one-third of the cases develop in extranodal sites, which have a heterogeneous histology with storiform and fascicular arrangement of plump spindle cells.^[24]^ The FDC tumors gained an increased attention since the first case of a tumor with follicular dendritic cell differentiation was reported by Monda et al^[25]^ in 1986. Up to now, there are <100 cases of follicular dendritic cell tumors which have been reported in English literature.^[12,25]^ The differential diagnosis include: sarcoma, Hodgkin disease, leiomyosarcoma, gastro-intestinal stromal tumor (GIST), and inflammatory pseudo-tumor.^[8,26,27]^ According to IHC analyses, FDC sarcoma shows spindle tumor cells arranging in fascicular or storiform patterns, which are immuno-reactive for one or more FDC markers: CD21 (C3d receptor, positive in 93% of cases) and CD35 (C3b receptor, positive in 89% of cases). Other quite specific markers used are: R4/23 (63%), Ki-67 (5–50%), EMA (41%), Vimentin (61%), HLA-DR (57%), CD45 (21%), and S-100 protein (31%). The tumor cells typically lack expression of CD1a, CD 68, and Desmin.^[12,14]^

Primary hepatic sarcomas are very rare, representing <0.1% of all primary hepatic tumors.^[7]^ Hepatic IPT-like FDC sarcoma is an extremely uncommon tumor and only 20 cases have been reported till now in English literature, including the present patient (summarized in Table 1).^[3,4,6–17]^ In 1996, Shek et al^[6]^ reported the first case of hepatic FDC sarcoma. The most common main complaint of the patients was upper abdominal pain and weight loss. Malaise, anemia, and fever were also part of the initial presentation in some number of patients. In 5 cases, patients were completely asymptomatic and the mass was found incidentally using contrast tomography scan and abdominal ultrasound. The age of all the patients at initial presentation ranged from 19 to 82 years (mean 47.8 years), the mean tumor diameter was 11.9 cm (3–20 cm), and the mean reported survival was >30 months (follow-up ranging from 6 to 108 months). The histology is similar to that of the conventional FDC tumor and it is generally considered to be a distinctive variant which is characteristically restricted to the abdomen and seems to be a separate clinico-pathologic entity. Comparing to the conventional FDC sarcoma, hepatic IPT-like FDC sarcomas have a marked female predominance (female to male ratio is 4:1), whereas conventional FDC sarcomas are not more prevalent in only one sex.^[1]^ Hepatic IPT-like FDC sarcomas have prominent inflammatory component, which makes them challenging to differentiate from inflammatory pseudo-tumors. IPT-like FDC sarcomas are strongly associated with the presence of EBV (85% in our review) infection, which is rare in conventional FDC sarcomas. Both the IPT-like FDC sarcomas and conventional FDC sarcomas generally show an indolent clinical behavior. Nevertheless, conventional hepatic FDC sarcomas can be more aggressive than IPT-like FDC sarcomas, and may recur or metastasize and even lead to death.

**Table 1:** Characteristics of patients with hepatic FDC sarcoma of the liver.

Case	Sex	Age, yr	Main complaint	Diameter, cm	EBV	Treatment	Recurrence/Survival	Published year
1	F	68	Malaise, weight loss, anemia	11	+	SR	No/>30m	1996^[4]^
2	F	35	Epigastric discomfort, fever, weight loss	20	+	SR	Yes/>30m	1996^[6]^
3	M	37	Malaise, weight loss, anemia	15	+	SR	No/>24m	1998^[7]^
4	F	19	Right upper quadrant pain, weight loss, palpable mass	12	+	SR	No/40m	2001^[3]^
5	F	56	Gastrointestinal discomfort	15	+	SR	Yes/NA	2001^[3]^
6	F	40	Epigastric pain, weight loss	12.5	+	SR	No/108m	2001^[3]^
7	F	49	Incidental at ultrasound	4.2	+	SR	No/9m	2001^[3]^
8	F	31	Abdominal distention, weight loss	15	+	SR	No/60m	2001^[3]^
9	F	57	Epigastric pain, weight loss	9.5	+	SR	No/36m	2001^[8]^
10	F	51	Epigastric pain, weight loss	12	+	SR	No/12m	2001^[8]^
11	M	82	Incidental on a CT abdomen	15	–	SR	No/18m	2005^[10]^
12	F	30	Incidental at ultrasound	5.5	+	SR	No/12m	2006^[9]^
13	F	57	Abdominal pain, vomiting, dizziness, liver dysfunction	13	+	SR	No/24m	2008^[14]^
14	F	78	Incidental at ultrasound	3	+	TACE	27m	2010^[11]^
15	F	59	Asymptomatic	6	+	SR	NA	2010^[17]^
16	F	53	Right upper quadrant pain, fever, anemia, jaundice	11.5	–	SR	No/6m	2011^[12]^
17	M	56	Right upper quadrant abdominal pain	11	NA	SR	No/12m	2011^[16]^
18	F	31	Palpable abdominal mass	20	+	SR	NA	2016^[13]^
19	M	19	Painless swellings around several joints	6	–	SR	NA	2016^[15]^

CT = contrast tomography, EBV = Epstein-Barr virus, FDC = follicular dendritic cell, m = month, NA = not available, SR = surgical resection, TACE = transcatheter arterial chemoembolization.

The initial diagnosis of FDCs is based on clinical examination, imaging, and pathologic assessment. The role of imaging is mainly in describing the extent of the mass and staging. When a mass is suspected in the liver, the confirmatory diagnosis of IPT-like FDC sarcomas is very difficult without pathological findings. It is noteworthy that the diagnosis of IPT-like FDC sarcomas should be based on the recognition of FDCs from microscopic findings. Actually, the distinction of hepatic IPT-like FDC sarcomas from other liver tumors is usually impossible without IHC results, due to features overlapping with other hepatic malignancies. Honestly, the patient was initial diagnosed as HCC or hepatic NHL according to his HBV infection's history, MRI appearance, and his liver resection history. Shek et al^[6]^ reported a case of hepatic IPT-FDC tumor which was initially misdiagnosed as an inflammatory pseudo-tumor. The tumor recurred 30 months after complete liver resection.^[6]^ The definite diagnosis of hepatic IPT-like sarcoma relies on histopathologic findings. Furthermore, CD21 and CD35 have been widely used as the preferred FDC markers which were expressed in almost IPT-like FDCs. Moreover, EBV infection was identified as a key role in the genesis of IPT-like FDC sarcomas.^[10]^ Almost all the hepatic IPT-like FDC sarcomas exhibited positive EBV-encoded small RNAs by in situ hybridization (17/20 in Table 1). LMP-1 gene, the major oncogene of EBV was identified in several cases of hepatic IPT-like FDC sarcomas.^[1,4]^ Positive staining of the FDC markers include CD21, CD35, and EBV-encoded small RNAs, while negative expression in CD23, SMA, Desmin, ALK1, and S-100 are in the present case. However, the pathogenic mechanism of EBV in IPT-like FDC sarcomas remains unclear and further investigation is required.

Primary NHL of the liver has rarely been reported and there are no typical laboratory or image diagnostic findings. Therefore, pathological analysis is the standard diagnostic method.^[28]^ The concurrence of hepatic NHL and IPT-like FDC sarcomas in the same patients has never been previously reported. The patient we reported in this article received hepatic segmentectomy in our hospital due to hepatic NHL (B-cell lymphoma) in 1999 (no data available due to long time). IHC studies demonstrated that all the tumor cells were strongly positive stainings with CD20 and CD45. A diagnosis of hepatic NHL was confirmed at that time (Figs. 1 and 2, Supplemental Digital Content). Several studies indicated that chronic hepatitis C virus (HCV) infection may be associated with the pathogenesis of NHL. However, the present case has HBV infection and no HCV infection. The detailed mechanism of HCV-mediated lymphomagenesis remains unclear.^[29]^ Our search of the literature found no such cases. This is the first report to demonstrate hepatic IPT-like FDC sarcoma in a patient with a history of primary hepatic NHL.

Surgical resection is an acceptable choice for primary hepatic IPT-like FDC sarcomas and hepatic NHL whenever possible. The efficacy of chemotherapy and radiotherapy is unclear. Daniel et al^[30]^ reported that even if complete resection had been achieved for hepatic NHL, postoperative chemotherapy was necessary. The patient in our report did not receive chemotherapy after the first hepatectomy. Tsunemine et al^[11]^ suggested that transarterial chemoembolization (TACE) was useful for the management of hepatic FDC sarcomas and the patient was still alive 27 months after the diagnosis of hepatic FDC sarcoma, which was favorably controlled by repeated TACE. Shinagare et al^[16]^ reported a case whereby the patient was not determined to be a surgical candidate due to large tumor mass and small residual liver volume. The patient received 4 cycles of standard-dose of chemotherapy comprising of cyclophosphamide, doxorubicin, vincristine, and prednisone therapy and portal vein embolization in an attempt to cause hypertrophy of the residual liver which made him a surgical candidate. Seven months later, the patient underwent a successful resection of the 12-cm nodular mass.^[16]^ In our review, 19 of the patients (95%) with hepatic IPT-FDC sarcomas only underwent partial hepatectomy and had a relative good long-term outcomes.

## 6 Conclusion

In conclusion, we reported a unique case of hepatic IPT-like FDC sarcoma with hepatic NHL history. IPT-like FDC sarcoma is receiving growing attention and the diagnosis may be correct with the aid of immuno-histochemical analysis being CD21 and CD35 the most reliable FDC markers. IPT-like FDC sarcoma should be considered in differential diagnosis when confronted with a liver tumor in a patient with primary NHL or HBV infection history. Complete resection remains the preferred method for the management of hepatic IPT-like FDC sarcomas which could get a better prognosis. In analyzing the reported cases, we brought forth differential diagnosis and provided evidence for further exploration of the pathogenesis of the tumor. Continued accumulation of characteristics of hepatic IPT-like FDC sarcomas would help in future patient care.

## Author contributions

**Conceptualization:** Er-lei Zhang.

**Data curation:** Jiang Li.

**Formal analysis:** Jiang Li, Hai-su Tao, Dong Chen.

**Funding acquisition:** Erlei Zhang.

**Investigation:** Jiang Li, Hai-su Tao.

**Methodology:** Zhiyong Huang.

**Project administration:** Erlei Zhang.

**Resources:** Jiang Li, Dong Chen, Hai-Su Tao.

**Supervision:** Er-lei Zhang, Zhiyong Huang.

**Writing – original draft:** Jiang Li.

**Writing – review & editing:** Er-lei Zhang, Jiang Li.

All authors read and approved the final manuscript.

## Supplementary Material

Supplemental Digital Content
