# Supplementary material for: Hepatic inflammatory pseudotumor-like follicular dendritic cell tumor with hepatic lymphoma history: A case report and literature review
Source: Medicine (Baltimore). 2021 Oct 1;100(39):e27392. doi: 10.1097/MD.0000000000027392 (PMC8483863; doi:10.1097/MD.0000000000027392)
Supplement: Supplemental Digital Content [file medi-100-e27392-s001.doc]

**Supplementary fig.1 and 2:** Surgical records (**Figure 1**) and pathological results (**Figure 2**) of the first operation due to Non-Hodgkin Lymphoma (B-cell Lymphoma) of the liver in 1999.
